# Mastication Wear of Two Low Profile Attachment Systems for Overdenture: An In Vitro Study

**DOI:** 10.1155/2022/6469890

**Published:** 2022-09-27

**Authors:** Alessandra Nicole Sassi, Claudia Todaro, Gaetano Isola, Ivano Bortolini, Ruggero Rodriguez y Baena, Stefano Storelli, Saturnino Marco Lupi

**Affiliations:** ^1^Department of Clinical Surgical, Diagnostic and Pediatric Sciences, University of Pavia, 27100 Pavia, Italy; ^2^Department of General Surgery and Surgical-Medical Specialties, University of Catania, Via S. Sofia 78, Catania 95124, Italy; ^3^Prosthodontic Lab, Via Driovilla 5, 31050 Miane, Italy; ^4^Department of Biomedical, Surgical and Dental Sciences, University of Milan, Milan, Italy

## Abstract

**Background:**

Edentulism is still a major problem in the world's population today. Implant-retained overdenture has proven to be a valid therapeutic solution in the mandible. This type of rehabilitation requires replacement of the matrices when those reach inadequate retention due to wearing processes. This study is aimed at evaluating the drop in retention of low-profile precision attachments following the application of vertical chewing forces. Two different attachment designs were compared.

**Methods:**

This in vitro study simulated an implant-retained overdenture on an edentulous mandible. Two low-profile attachments were compared. Loaded and unloaded sides were considered. Tests were performed by exerting a vertical cyclic force on the prosthesis at the level of the first molar of a hemiarch. Retention tests were performed before and after 400.000 chewing cycles, comparable to one year of use.

**Results:**

The presence of vertical load wear was identified and characterized. Retention never fell below the values indicated by the manufacturer. Furthermore, significant differences were identified between the two retention systems.

**Conclusions:**

Loss of occlusal load retention is a component that must be evaluated by the clinician during the design of implant-prosthetic rehabilitation, particularly in those cases where elevated occlusal forces or parafunctions are present.

## 1. Introduction

The loss of dental apparatus directly leads to a reduction in masticatory, phonatory, and aesthetic capacities, with consequences on the patient's general and psychological health [1]. Although the prevalence of total edentulism has decreased significantly in recent years, the increase in average life expectancy in industrialized countries has increased the number of people needing complete prosthetic rehabilitation. Furthermore, in contrast, in developing countries with lower life expectancies, total edentulism is more common and begins at an early age [2–4]. Edentulism has a significant effect on residual crestal resorption, loss of vertical dimension, and soft tissue alterations with the consequent onset of diseases such as angular cheilitis, stomatitis, and oral candidiasis [5, 6]. The loss of periodontal structures and receptors due to the reduction in the number of teeth and the chewing surface is associated with impaired chewing and lower chewing forces [7]. This can lead to altered food choices with a negative impact on the diet and consequent nutrient intake [8, 9]. The same pathologies that lead to dental loss, such as carious and periodontal diseases, consist of chronic infections, which are associated with increased inflammatory state [10]. These conditions can lead to an increased incidence of hypertension and cardiovascular disease [11]. These deleterious consequences on oral and general health clarify the importance of taking care of residual dental elements and the rehabilitation of the edentulous areas.

There are many options available to ensure a physiological dentition, such as traditional dentures, implant-supported fixed prosthesis, and implant-retained removable overdenture [12–17]. Traditional dentures are no longer considered an adequate therapeutic choice [14, 18]. Furthermore, the workflow has been modified with the introduction of digital processes such as decisional software, intraoral and extraoral scanners, and CAD-CAM technologies [19]. The decision-making process is dependent on clinical evaluations such as bone loss, soft tissue conditions, and general health. However, the patient's expectations and financial budget must also be taken into account [20, 21].

Overdenture has numerous benefits that make it a first-choice treatment option, particularly for the lower mandible rehabilitation [15, 18, 22, 23]. A fixed prosthesis not only implies higher costs but also prolonged surgical times and more demanding surgeries for the patient. The placement of fewer implants results in a simplified procedure with reduced risks associated to surgery and anesthesia, especially in problematic and/or multipathological patients [24].

To ensure a successful rehabilitation in the long run, it is also necessary to perform appropriate hygiene maneuvers [25, 26]. The possibility of removing the overdenture also allows for a simpler procedure in less motivated or less capable patients [27, 28].

Because of these characteristics, the literature investigating overdenture has increased in the past years. Several studies have evaluated the correlation between the retention strength of the attachments and the stability of the prosthesis, the quality of masticatory function, and the patient satisfaction [13, 29–31]. Different research focused on the characteristics of attachments, such as material, design, and dimensions. Most studies evaluated the retention properties of overdenture attachments by measuring wear due to axial insertion and removal cycles. However, in clinical applications, it is well known that true forces are always tridimensional. The wear and retention forces detected can also be influenced by the implants tilting, the mastication cycle as well as the technique of removal of the overdenture [32, 33].

A direct relationship between prosthesis retention and patient satisfaction has been reported. Retention is defined as the resistance to displacement forces. According to some researchers, patient satisfaction appears to be higher in rehabilitations with splinted attachments. On the other hand, ball or ball-like attachments seem to lead to smaller bending moments and are also easier to place and maintain [34–36].

Nevertheless, literature does not provide clear indications on which is the best overdenture retention system, but rather pros and cons are listed. Currently, the choice of attachment is mainly based on the experience of the clinician, rather than on indications of the scientific literature.

Our study concentrated on wear due to masticatory forces, neglecting the insertion and removal wear.

Retention tests were performed considering different parameters affecting the attachment performance. Two types of low-profile precision attachments have been considered. The different behaviors of loaded and nonloaded attacks were also compared.

This study is aimed at quantifying the differences in the performance of two low-profile precision attachments and between two loading settings.

## 2. Materials and Methods

### 2.1. Sample Preparation

In this in vitro study, two low-profile attachment systems were compared: Group A using Ot-Equator® (Rhein 83 Srl, Bologna, Italy) and Group B using Locators® (Zest Anchors, LLC, Carlsbad, CA, USA). Each attachment system is composed of a matrix (i.e. the portion of an attachment system that receives the male [37]) and a patrix (i.e. the extension of a dental attachment system that fits into the recessed extension of the attachment, the matrix [37]).

**Figure 1:**
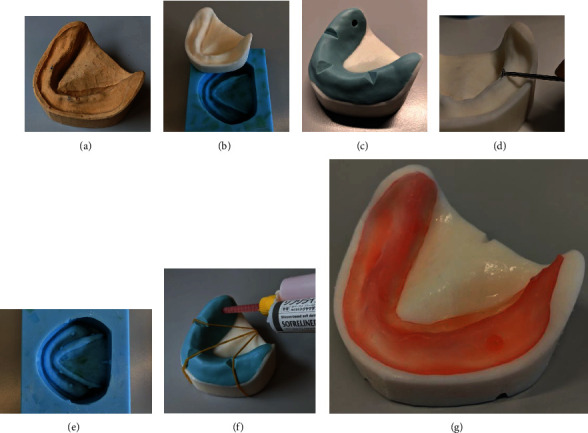
(a) Plaster model of the edentulous mandible; (b) silicone mold and epoxy resin duplicate; (c) silicone mask; (d) homogeneous reduction of the surface; (e) duplication silicone mask; (f) silicone injection; (g) final specimen of the edentulous mandible.

To perform the experiments, one specimen was realized for each group as described below. A silicone mask (Vestige Duple 24”, Trayart srl, Castelbaldo, PD, Italy) was obtained starting form a plaster model of an edentulous mandible and used to make one epoxy resin model (UP370 L, Trias Chem srl, Torrile, PR, Italy) (Figures 1(a)–1(b)). Another silicone mask of the ridge was also realized (Figure 1(c)). A homogeneous layer of 1,5 mm material was removed from the surface of the epoxy resin model using a pilot bur (806 104 304524 050, KOMET ITALIA Srl, Milan, Italy)(Figure 1(d)). To reach a better stability of the pseudo-gingiva, a few holes were also made with a round bur (310 104 001001 042, KOMET ITALIA Srl, Milan, Italy). The milled epoxy resin model was duplicated thanks to another silicone matrix (HD Sil 80, Techim Group srl, Arese, MI, Italy) to obtain two identical specimens (IPU 812, Trias Chem srl, Torrile, PR, Italy) (Figure 1(e)). Silicone (Tokuyama Sofreliner Tough M, Encinitas, California, USA) was injected through two holes realized in the silicon mask, stabilized on the epoxy resin model (Figure 1(f)). The simulated gingiva obtained was refined and placed on the model after full polymerization was obtained (Figure 1(g)).

One denture for each group was made with acrylic resin (Aesthetic blue F34, Candulor, Glattpark, Opfikon, Switzerland). Teeth (NFC+, Candulor, Glattpark, Opfikon, Switzerland) were mounted on the center of the edentulous ridge according to Gerber's technique [38].

**Figure 2:**
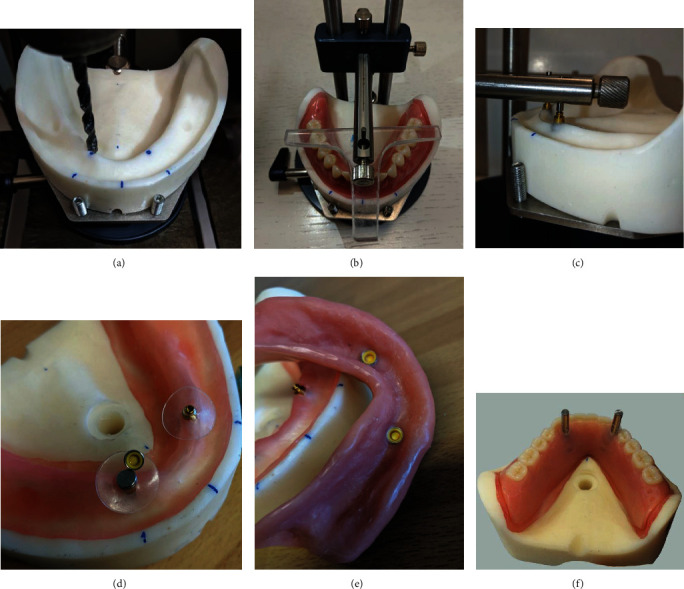
(a) Preparation of the site in the canine region; (b) visualization of the occlusal plane; (c) solidarization of the implant analogues with liquid resin; (d) plastic discs placed to protect the model and stainless-steel retentive caps, housing the retentive inserts, positioned; (e) finished overdenture; (f) pivots.

Two Zimmer implant replicas Ø 3.5 mm (Zimmer Biomet Dental, Palm Beach Gardens, FL, USA) were positioned in the epoxy resin models in the canine region (Figure 2(a)). The occlusal plane of the dentures was visualized and used to place the implants perpendicularly (Figure 2(b)). The replicas and the screwed attachments were solidarized to the model using liquid resin (Figure 2(c)).

Stainless-steel retentive caps, housing the retentive inserts, were positioned on top of the attachments, previously isolated with plastic discs (Figure 2(d)). Those were later embedded in the dentures in the appropriate position (Figure 2(e)). Two pivots were also placed above the attachments, on the occlusal plane, to allow a stable grip during the following retention tests (Figure 2(f)).

### 2.2. Experimental Tests

MTS Acumen® (MTS® spa, Eden Prairie, MN, USA) was used to emulate the masticatory cycles.

**Figure 3:**
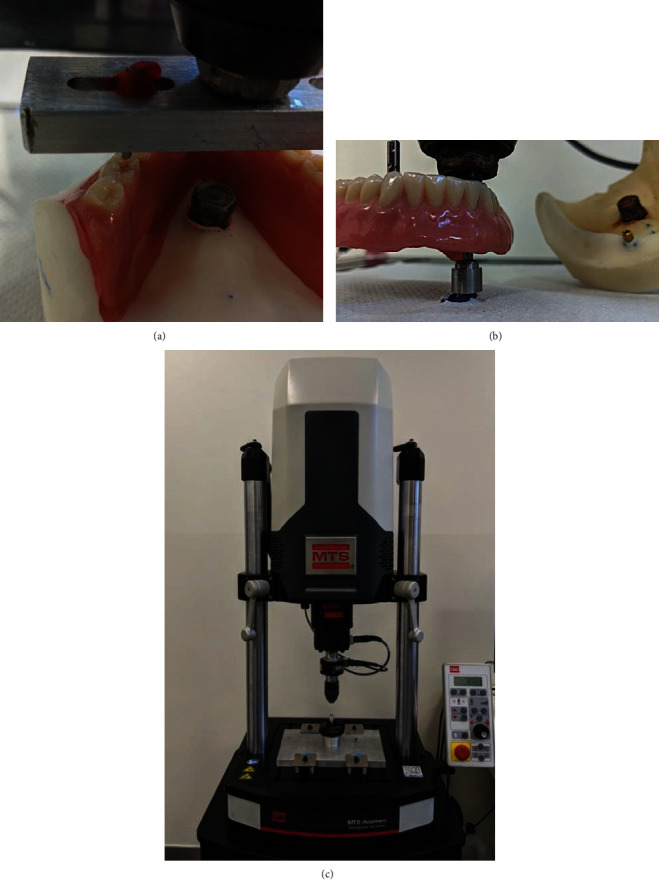
A) Engagement of the first molar; B) Retention test setting; C) MTS Acumen.

Masticatory forces were applied on one side of the denture, considering that natural masticatory cycles are executed on one hemimandible at a time [39]. The masticatory forces were simulated through a pivot engaging the first molar, perpendicularly to the occlusal plane (Figure 3(a)). The occlusal pattern was tripodic and was located in the central groove.

These tests were performed in Group A, with pink (medium retention) and clear (high retention) polyamide retentive inserts, and in Group B, with pink (medium retention) and clear (high retention) polyamide retentive inserts.

The matrix retention forces, as declared by the manufacturer, are reported in Table 1 [40, 41].

While the cycles were running, the samples were placed in artificial saliva (SINOPIA sas, Torino, Italy), at a temperature of 37° C. The masticatory force was 250 N with a frequency of 1.6 Hz.

Every 400,000 masticatory cycles, the retentive inserts (matrix) were replaced and retention tests were performed before and after this replacement. Overall, the test involved 1,200,000 masticatory cycles, corresponding to 3 years of use.

To measure the retentive capacity of the single attachment, the patrices were disassembled from the model, screwed individually on a support and tested every 400,000 cycles. The dentures were engaged by a punch, in correspondence with the pivot related to the attachment under study (Figure 3(b)). The punch was connected to the load cell of an electrodynamic tensile testing machine MTS Acumen® (Figure 3(c)). The testing machine was used to remove the denture ten times vertically and uniaxially, evaluating the maximum force needed to remove the prosthesis from the attachment system. Ten insertion-removal cycles were executed to have enough data to compute a correct mean.

**Figure 4:**
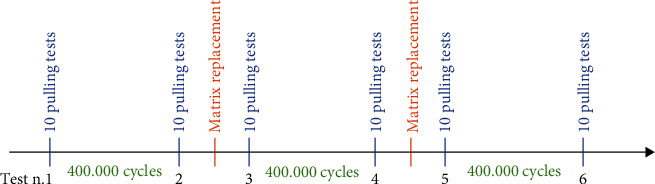
Timeline of the masticatory cycles and tests.

Finally, the data we hold was relative to ten retention values for each attachment, right and left, for each setting, before and after the replacement of the matrices, and after 400,000, 800,000 and 1,200,000 cycles (Figure 4).

### 2.3. Data Collection and Analysis

Two factors were separately analyzed by comparing datasets by pairs:

- The shape of the attachment system and the different material of the retentive inserts, comparing groups A and B
-The loading setting, comparing the attachment on the side of the mastication and the one on the opposite side

To evaluate the drop in retention due to the matrix and the one due to the wear of the patrix separately, different evaluations have been made for each phase for both samples of the pair. To evaluate the wear of the matrices, the comparison was made on the difference between the values of tests done before and after the 400,000 cycles. To evaluate the patrix wear, the differences between the results of the test done with new retentive inserts every time were compared. The list of comparison is presented in Table 2.

### 2.4. Statistical Analysis

The mean and the standard deviation for each series of 10 pulling tests were calculated. This allowed to characterize the real value with confidence and to have a statistical indicator about the uncertainty of the measure. The Student's t test was used to compare two series of results, for example to compare the results of groups A and B. The significance level was set at 0.05.

## 3. Results

Measurement results are reported in Table 3, and Table 4.

**Figure 5:**
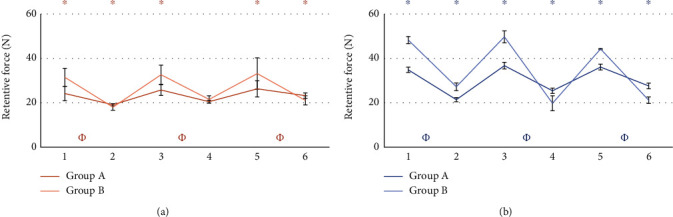
Comparison between loaded attachments from Group A and B. Tests using pink matrix (a) and tests using clear matrix (b). ^∗^: statistically significant difference between retention forces; *Φ*: statistically significant difference between retention loss after 400.000 masticatory cycles.

**Figure 6:**
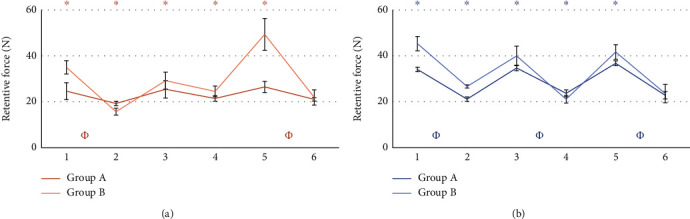
Comparison between unloaded attachments form Group A and B. Tests using pink matrix (a) and tests using clear matrix (b). ^∗^: statistically significant difference between retention forces; *Φ*: statistically significant difference between retention loss after 400.000 masticatory cycles.

**Figure 7:**
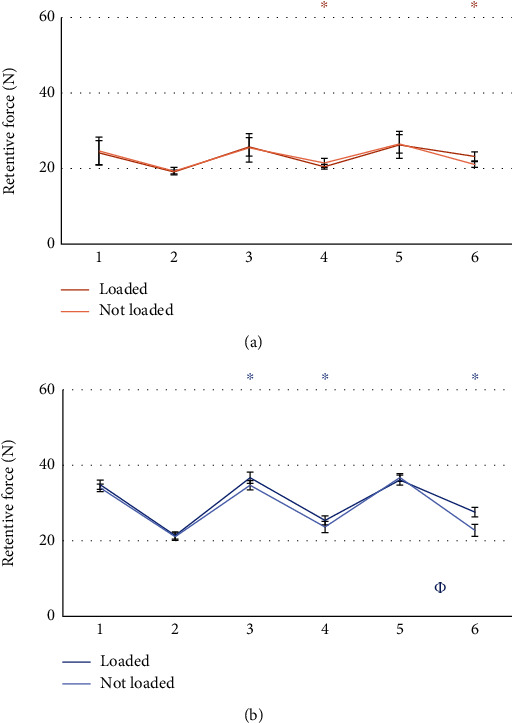
Comparison between loaded and unloaded attachments form Group A. Tests using pink matrix (a) and tests using clear matrix (b). ^∗^: statistically significant difference between retention forces; *Φ*: statistically significant difference between retention loss after 400.000 masticatory cycles.

**Figure 8:**
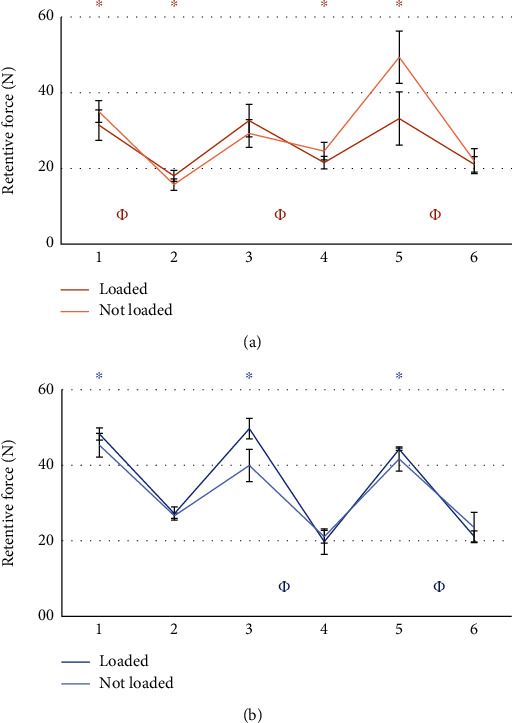
Comparison between loaded and unloaded attachments of Group B. Tests using pink matrix (a) and tests using clear matrix (b). ^∗^: statistically significant difference between retention forces; *Φ*: Statistically significant difference between retention loss after 400.000 masticatory cycles.

**Figure 9:**
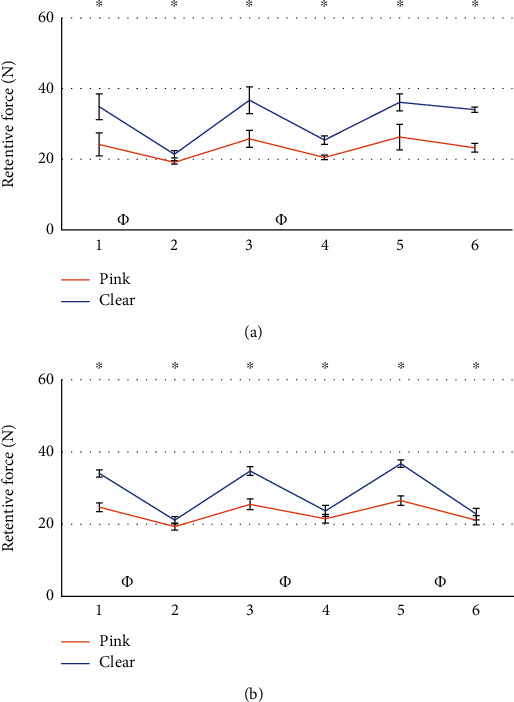
Comparison between pink and clear matrices in group A. Tests performed on the loaded side (a), and tests performed on the unloaded side (b). ∗: statistically significant difference between retention forces; *Φ*: Statistically significant difference between retention loss after 400.000 masticatory cycles.

**Figure 10:**
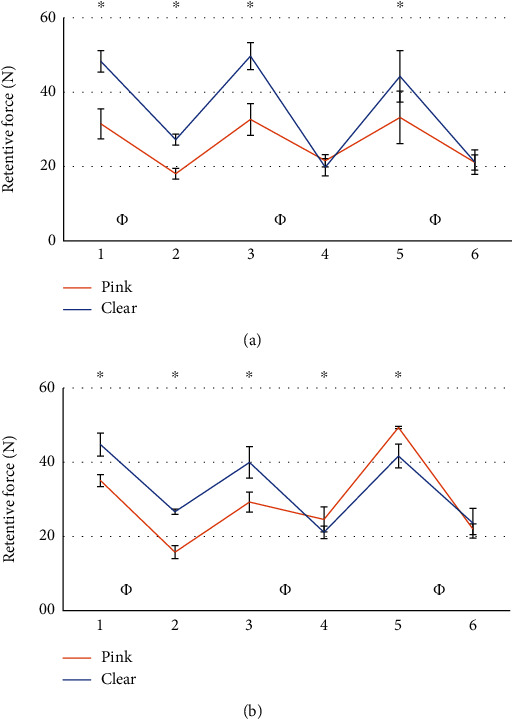
Comparison between pink and clear matrices from Group B. Tests performed on the loaded side (a), and tests performed on the unloaded side (b). ^∗^: statistically significant difference between retention forces; *Φ*: Statistically significant difference between retention loss after 400.000 masticatory cycles.

The comparison between the retention value of the patrices before and after the masticatory cycles did not give statistically significant results. Results from the statistical analysis relatives to the wear of matrices are reported in Figures 5–10.

### 3.1. Group A vs Group B

### 3.2. Loaded vs Unloaded Sides

### 3.3. Pink vs Clear Matrices

## 4. Discussion

In this work, the loss of retention between two different low-profile precision attachments for overdentures was evaluated. Although several studies are present in the literature evaluating the loss of retention due to the insertion and disinsertion of the prosthesis [42–44] in the present study, the loss of retention due to mastication was investigated.

To the best knowledge of the authors, this is the first investigation of this kind, using a full prosthesis to simulate realistic conditions. The wear due to mastication, which produces the loss of retention, was isolated from other possible influencing factors. All the attachments, regardless of the loading condition or their type, showed retention within suggested limits during all the tests. In fact, several authors pointed out that value of 8-10 N are optimal, while others suggested 20 N [36, 45]. In both cases, the attachments examined in this work appeared to be suitable, because both attachments maintained a retention force above the minimum recommended level. However, the latter statement should be taken with caution, as no wear due to insertion was considered. New matrices exhibited values of retention significantly higher than those suggested above, while matrices after 400,000 cycles had values close to 20 N. Almost in all cases, the retention declared by the manufacturer was always respected. However, in one test in Group B after 400,000 cycles, values lower than the declared one were observed. This result must be interpreted with caution as, although not significant, it only represents the loss of retention due to vertical load and in clinical use at least the loss of retention due to removal of the prosthesis must be added to it. Clinically, this could lead to insufficient retention.

### 4.1. Group A vs Group B

Several comparisons have been made between all data available. To compare the two systems, comparisons have been performed considering attachments of the same side with respect to the point of application of the force, and with the most similar matrix, as from the manufacturer datasheet (Table 1). It was possible to observe the wear of the matrix in all tests. The retention loss of Group A has always been significantly lower than that of Group B. In some cases, while Group B exhibited a higher retention at the beginning of life, it was found to have a lower retention with respect to Group A after 400,000 cycles (Figures 5 and 6). A possible explanation is that because of greater retentive forces, resulting from higher contact surfaces and higher frictional forces, greater and faster wear follows [45, 46]. To corroborate this hypothesis, comparisons between matrices with different retention levels have been evaluated. Clear matrices, with higher retention levels declared by the manufacturer for both Groups A and B, were subjected to greater loss after 400.000 mastication cycles (Figures 9 and 10). The pink matrix group showed a higher retention level only during tests n.5 of the unloaded Group B, perhaps because of variability during the placement of the matrices. However, higher initial values were still followed by greater losses, proving our hypothesis. In conclusion, in Group A, an initially lower but then more constant retention force was observed.

### 4.2. Loaded Side vs Unloaded Side

Another important comparison was between the two possible loading schemes for the attachments: loaded and unloaded attachments. The experiments considered attachments working simultaneously on the same model, therefore, with the same type and the same matrix (Figures 7 and 8). The results between these comparisons were not easy to interpret. First, while they were statistically significant, in some cases they favored the load side and in other case the not-load side. Moreover, their magnitude was relatively small. Therefore, from a practical point of view, they might not be significant. Furthermore, after 400,000 cycles, Group A exhibited higher retention from the loaded side with pink matrix in test 6 and with clear matrix in tests 4 and 6. However, a lower retention of the loaded side was measured with the pink matrix in test 4. The difference in retention was statistically significant only on one set of measurements for the clear matrix. Group B, on the other hand, demonstrated a loss of retention in two tests with clear matrix. These observations may suggest a higher loss of retention for the loaded side of Group B and for the unloaded side of Group A.

Furthermore, after 400,000 cycles, Group A exhibited higher retention from the loaded side with pink matrix in test 6 and with clear matrix in tests 4 and 6. However, a lower retention of the loaded side was measured with the pink matrix in test 4. The difference in retention was statistically significant only on one test for the clear matrix.

Group B similarly demonstrated a significant difference, sometimes in favor and sometimes against the loaded side, either after masticatory cycles or considering loss of retention. However, due to the low entity of the phenomena, and the possible uncertainty given by the matrix substitution, clearly indicated by the variability measured before the execution of the 400,000 cycles, the observations are not conclusive. Indeed, it seems impossible to extract a general rule from the data.

The limitations of this study are primarily that it is an in vitro study in which the masticatory cycles were administered in a standardized manner. Chewing in the real population presents complex and different patterns. The aim of this study, however, was to investigate the possibility of wear due to masticatory forces.

Another limitation is represented by the inherent error in the replacement of matrices, which has determined a variability in the results that is difficult to eliminate.

## 5. Conclusions

This work investigated the loss of retention in two overdenture systems due to masticatory wear. This loss was clearly observed and measured for both systems. Different behaviors were obtained for the two tested systems. The differences between attachment systems should be considered during diagnosis and treatment planning.

Further work is required to investigate different masticatory forces on the systems, to understand how this affects the behavior of the systems.

## Figures and Tables

**Table 1:** Matrix retention declared by the manufacturer.

Color/retention level	Retention	
	Group A	Group B
Pink/medium retention	11.768 N	13.345 N
Clear/high retention	17.652 N	22.241 N

**Table 2:** List of Comparison.

Wear of the matrices	Test n. 2 - test n. 1	Test n. 4 - test n. 3	Test n. 6 - test n. 5
Comparison of Wear of the patrix	Test n. 3 vs test n. 1		
	Test n. 5 vs test n. 1		
Comparison of wear of the matrices	Wear of the matrices in group A vs Wear of the matrices in group B – Loaded side (Figure 5)		
	Wear of the matrices in group A vs Wear of the matrices in group B – Unloaded side (Figure 6)		
	Wear of the matrices in loaded side vs Wear of the matrices in unloaded side – Group A (Figure 7)		
	Wear of the matrices in loaded side vs Wear of the matrices in unloaded side – Group B (Figure 8)		
	Wear of the pink matrices vs Wear of the clear matrices – Group A (Figure 9)		
	Wear of the pink matrices vs Wear of the clear matrices – Group B (Figure 10)		

**(a):** 

		Test *N*°	1	2	Difference between 1 and 2
		Matrix	0 cycles	400.000 cycles	
Group	Attachment	Patrix	0 cycles	400.000 cycles	
A	L	Mean ± SD	24.1748 ± 3.2646	19.1077 ± 0.5004	-20.96%
A	NL		24.6844 ± 3.6536	19.3199 ± 0.9977	-21.73%
B	L		31.4685 ± 4.0374	18.0411 ± 1.4338	-42.67%
B	NL		35.0551 ± 2.875	15.746 ± 1.501	-55.08%

**(b):** 

		Test *N*°	3	4	Difference between 3 and 4
		Matrix	0 cycles	400.000 cycles	
Group	Attachment	Patrix	400.000 cycles	800.000 cycles	
A	L	Mean ± SD	25.7573 ± 2.4303	20.4907 ± 0.6287	-20.45%
A	NL		25.4889 ± 3.789	21.4927 ± 1.1888	-15.68%
B	L		32.649 ± 4.2927	21.5418 ± 1.6351	-34.02%
B	NL		29.2533 ± 3.6557	24.5668 ± 2.3136	-16.02%

**(c):** 

		Test *N*°	5	6	Difference between 5 and 6
		Matrix	0 cycles	400.000 cycles	
Group	Attachment	Patrix	800.000 cycles	1.200.000 cycles	
A	L	Mean ± SD	26.2802 ± 3.5966	23.2298 ± 1.2188	-11.61%
A	NL		26.5362 ± 2.4276	21.0904 ± 0.7476	-20.52%
B	L		33.178 ± 7.0549	21.0805 ± 2.0535	-36.46%
B	NL		49.3771 ± 6.9115	21.941 ± 3.3065	-55.56%

L: loaded side. NL: non-loaded side. All data, except for differences expressed as a percentage, are expressed in Newtons.

**(a):** 

		Test *N*°	1	2	Difference between 1 and 2
		Matrix	0 cycles	400.000 cycles	
Group	Attachment	Patrix	0 cycles	400.000 cycles	
A	L	Mean ± SD	34.8522 ± 1.2194	21.3934 ± 0.9581	-38.62%
A	NL		34.052 ± 0.9946	21.1054 ± 0.9969	-38.02%
B	L		48.27 ± 1.6027	27.2141 ± 1.7513	-43.62%
B	NL		45.2969 ± 3.1341	26.6368 ± 0.7034	-41.20%

**(b):** 

		Test *N*°	3	4	Difference between 3 and 4
		Matrix	0 cycles	400.000 cycles	
Group	Attachment	Patrix	400.000 cycles	800.000 cycles	
A	L	Mean ± SD	36.7098 ± 1.5028	25.4121 ± 1.2228	-30.78%
A	NL		34.7251 ± 1.2019	23.6696 ± 1.5049	-31.84%
B	L		49.7079 ± 2.7206	19.7881 ± 3.3769	-60.19%
B	NL		39.9586 ± 4.2645	21.0962 ± 1.6861	-47.20%

**(c):** 

		Test *N*°	5	6	Difference between 5 and 6
		Matrix	0 cycles	400.000 cycles	
Group	Attachment	Patrix	800.000 cycles	1.200.000 cycles	
A	L	Mean ± SD	36.0954 ± 1.3159	27.6003 ± 1.2681	-23.54%
A	NL		36.7553 ± 1.0438	22.7764 ± 1.5634	-38.03%
B	L		44.2459 ± 0.3149	21.1758 ± 1.4459	-52.14%
B	NL		41.6631 ± 3.1943	23.5504 ± 4.0025	-43.47%

L: loaded side. NL: nonloaded side. All data, except for differences expressed as a percentage, are expressed in Newtons.

## Data Availability

The data used to support the findings of this study are available from the corresponding author upon request.
